# Response selection modulates crowding: a cautionary tale for invoking top-down explanations

**DOI:** 10.3758/s13414-019-01891-5

**Published:** 2019-12-03

**Authors:** Josephine Reuther, Ramakrishna Chakravarthi

**Affiliations:** University of Aberdeen, Aberdeen, UK

**Keywords:** Object recognition, Crowding, Knowledge, Similarity, Top-down, Bottom-up, Response selection

## Abstract

Object recognition in the periphery is limited by clutter. This phenomenon of *visual crowding* is ameliorated when the objects are dissimilar. This effect of inter-object similarity has been extensively studied for low-level features and is thought to reflect bottom-up processes. Recently, crowding was also found to be reduced when objects belonged to explicitly distinct groups; that is, crowding was weak when they had low group membership similarity. It has been claimed that top-down knowledge is necessary to explain this effect of group membership, implying that the effect of similarity on crowding cannot be a purely bottom-up process. We tested the claim that the effect of group membership relies on knowledge in two experiments and found that neither explicit knowledge about differences in group membership nor the possibility of acquiring knowledge about target identities is necessary to produce the effects. These results suggest that top-down processes need not be invoked to explain the effect of group membership. Instead, we suggest that differences in flanker reportability that emerge from the differences in group membership are the source of the effect. That is, when targets and flankers are sampled from distinct groups, flankers cannot be inadvertently reported, leading to fewer errors and hence weaker crowding. Further, we argue that this effect arises at the stage of response selection. This conclusion is well supported by an analytical model based on these principles. We conclude that previously observed effects in crowding attributed to top-down or higher level processes might instead be due to post-perceptual response selection strategies.

**Electronic supplementary material:**

The online version of this article (10.3758/s13414-019-01891-5) contains supplementary material, which is available to authorized users.

## Introduction

Visual crowding is the dramatic breakdown of object recognition in the presence of nearby objects, and has been studied extensively over the past 50 years (e.g. Bouma (1970), Flom et al., (1963), Levi (2008), and Strasburger and Malania (2013)). It is ubiquitous (Levi, 2008; Whitney & Levi, 2011) and provides a natural window into object recognition mechanisms by highlighting the constraints under which successful recognition occurs. Several theories have been proposed to account for the inter-object interference observed in crowding. A prominent hypothesis suggests that it is a limitation of bottom-up processes involved in feature integration (e.g. Greenwood et al., (2009), Harrison and Bex (2017), Parkes et al., (2001), and Pelli et al., (2004)). According to this account, when an object is presented in isolation or far from other objects, its features are detected and appropriately combined to produce a recognisable object representation. However, when two or more objects are located too close to each other, features from multiple nearby objects are inappropriately integrated into a single representation, leading to an inability to identify the objects - crowding. Other accounts include substitution or mislocation errors (e.g. Chung and Legge (2009), Hanus and Vul (2013), Nandy and Tjan (2007), Strasburger and Malania (2013), Strasburger (2005), and Zhang et al., (2012)), where the positions of objects are mistaken, or a lack of attentional resolution (He et al., 1996; Strasburger, 2005; Treisman and Gelade, 1980), where selective attention cannot individuate a given object exclusively when other objects are too close to it.

The main factor that determines crowding is inter-object spacing, which has been investigated for a wide range of stimuli from gabors and letters to faces and furniture (Pelli & Tillman, 2008; Wallace & Tjan, 2011). The closer the flankers are to the to-be-identified object (target), the greater the impairment in identification. The minimum spacing beyond which the presence of additional objects does not interfere with the correct recognition of a target is known as critical spacing. It has been well established that, under most circumstances, critical spacing depends on target-eccentricity and scales with it (Bouma, 1970; Pelli & Tillman, 2008; Wallace & Tjan, 2011).

There is considerable evidence that crowding is also modulated by the similarity between a target and its flankers. For example, the strength of crowding is affected by low-level differences in properties such as contrast polarity (Chakravarthi & Cavanagh, 2007; Kooi et al., 1994), colour (Kennedy & Whitaker, 2010; Põder & Wagemans, 2007), shape (Freeman et al., 2012; Kooi et al., 1994; Nazir, 1992), spatial frequency (Chung et al., 2001) and complexity (Zhang et al., 2009). It also seems to be modulated by differences in object category (Huckauf et al., 1999; Reuther & Chakravarthi, 2014) and level of processing (features, object parts or holistically, e.g. Farzin et al., (2009), Ikeda et al., (2013), Louie et al., (2007), and Martelli et al., (2005)). (but see Kalpadakis-Smith et al., (2018)) Crowding is stronger when target and flankers share the same properties, but weaker when they do not. For instance, target identification was worse when both target and flankers had the same complexity, compared to when either the target or the flankers had a different complexity (Zhang et al., 2009).

Object similarity is thought to modulate crowding through bottom-up processes, such as mutual inhibition between objects that share features (Kooi et al., 1994), competition between similar features for feature integration (Bernard and Chung, 2011; Freeman et al., 2012), spatial uncertainty of shared features leading to misbinding of confusable features (Chung & Legge, 2009) or an inability to select a non-salient object (Chakravarthi & Cavanagh, 2007). On the other hand, recent studies have argued that top-down influences play a substantial role in the effect of similarity on crowding. One proposal that supports this position is that grouping modulates crowding (e.g. Herzog et al., 2015, Manassi et al., 2012; Manassi & Whitney 2018). Since grouping is strongly modulated by Gestalt properties such as similarity, the latter should affect crowding. That is, similar objects group together into a single whole, which makes it hard to identify the individual elements - crowding; on the other hand, dissimilar objects do not group together, allowing the target to be individuated, hence alleviating crowding. This grouping process can be argued to be a top-down process implying that the effect of similarity on crowding acts through top-down processes. But note that even in this proposal, the cues for grouping are low-level featural differences. Further, and more importantly, it has been posited that grouping by Gestalt properties is driven by bottom-up processes (Roelfsema & Houtkamp, 2011). Hence the effect of similarity, even if mediated by grouping, could still be a bottom-up process.

Zhang et al., (2009) invoked higher-level processes, in addition to bottom-up mechanisms, to account for their findings on complexity differences (objects of similar complexity crowd each other more than objects of dissimilar complexity). They proposed that top-down explicit knowledge of whether targets and flankers belong to the same group (as when both targets and flankers have the same complexity) or were sampled from different sets (as when they do not have the same complexity) influences crowding. In their study, when the objects differed in complexity, say a simple target surrounded by complex flankers, the complex characters would never be response options. Participants could use this knowledge of complexity differences to guide the individuation of the simple target or to ignore any complex flanker they might have identified. However, this would not be the case when the targets and flankers were of the same complexity (say a simple target surrounded by simple flankers). Here, individuation based on complexity is not possible and further, any of the identified objects, including the flankers, would be reportable. Similarly, knowledge about possible target identities could be used by the visual system to reject interpretations of the feature-integration output that do not fit with any of the known response options (Bernard & Chung, 2011).

To test their top-down proposal that crowding between featurally dissimilar groups of objects is modulated by top-down effects of knowledge in addition to bottom-up effects, Zhang et al., (2009) equated stimulus complexity and tested the effect of observers’ knowledge of target identities in isolation. They found that crowding was stronger when targets and flankers were sampled from the same set, than when they were selected from different sets. In this experiment, participants were explicitly informed that in blocks where targets and flankers were drawn from different sets, the flankers would never be possible response options. They were also informed whether the targets and flankers would be sampled from the same set or from different sets at the beginning of each block. Based on this, the authors concluded that participants used their knowledge about flanker and target sets to alleviate the effect of crowding. They argued that top-down knowledge either prevented target-flanker mislocalisation or facilitated target-flanker segregation when targets and flankers were selected from distinct sets, and hence modulated object recognition. Such mechanisms were claimed to act in addition to bottom-up mechanisms that exploited low-level differences when objects are dissimilar. This argument can be extended to other established effects of similarity (colour, shape, spatial frequency, etc.). Hence, such results might suggest that both bottom-up and top-down processes play an important role in the effect of similarity on crowding. Further, importantly, note that the authors argue for perceptual effects (reduced target-flanker interference) as a result of feedback processes. However, it is quite possible that, in addition to bottom-up processes, additional differences arise purely at the response or decision stage, without the need to invoke any top-down feedback processes.

Determining which of these possibilities is being implemented by the visual system in addition to processes that are based on low-level featural differences also has implications for certain claimed higher-level effects in crowding. One such example is the effect of object category. Similarity in category membership of target and flankers has been shown to modulate crowding. That is, crowding is stronger when target and flankers are from the same category (e.g. target and flankers are all letters) than when the flankers belong to a different category, and this effect (although reduced considerably) is still observed when featural differences are equated for (e.g. the target is a letter, but the flankers are numbers; Huckauf et al., 1999; Reuther & Chakravarthi 2014). These and similar findings have been taken to argue that crowding affects object recognition at different levels of processing, including the level where category membership is assigned or the level where faces are holistically processed (Whitney & Levi, 2011). However, in light of Zhang et al.,’s findings, it can be argued that participants could simply have used their knowledge about category membership of the target and knowledge about the possible target identities to reduce inter-object interference. This knowledge could modulate crowding at a perceptual stage (as Zhang et al., argue) or at a post-perceptual decision stage (as we will argue). If it is the latter, target-flanker interactions might not be occurring at multiple levels, as has been claimed by several recent studies (e.g. Farzin et al., 2009; Kimchi & Pirkner 2015; Louie et al., 2007; Whitney & Levi), but might take place only at the feature integration level, with post-perceptual decisions contributing to differences in performance.

In two experiments, we will test the proposal that explicit knowledge is not necessary to explain the findings from previous studies. In particular, implicit consequences of differences in target- and flanker-sampling (from the same of from different stimulus-sets), without the need for explicit knowledge, should be sufficient to lead to differences in crowding similar to those reported by Zhang et al., (2009). In the first experiment, we will manipulate the extent of overlap between target and flanker sets, while not informing participants of such differences to determine if the crowding effects observed by Zhang et al., can be replicated in the absence of top-down knowledge. In this experiment, we will also test, if these differences in overlap can explain the reported effect of target-flanker category. In the second experiment, we will introduce a condition where top-down knowledge is impossible to develop and sustain, and check if modulating effects on crowding are still observable. Finally, we will model our and Zhang et al.,’s data using a simple bottom-up model, without any top-down knowledge, to examine if such a model can capture observers’ performance.

## Experiment 1: Overlap effects without explicit knowledge

Zhang et al., (2009) demonstrated that providing participants with knowledge about possible target identities (response options) and differences in stimulus sampling (flankers drawn from the target-set or from a different set) modulated crowding. In the same vein, differences in target and flanker sampling can be unwittingly introduced in experiments investigating effects of target-flanker-similarity at ‘higher’ levels of processing, such as the stages where familiarity or category are processed (Huckauf et al., 1999; Reuther & Chakravarthi, 2014). However, in these latter experiments participants were usually not made aware of the existence of different target-flanker sampling conditions. Nevertheless, it is possible that participants gained such knowledge over the course of the experiment, which could have driven the claimed higher-level effects, in addition to the influence of low-level feature similarity (Reuther & Chakravarthi, 2014). On the other hand, overlap between target and flanker sets might modulate crowding even in the absence of top-down knowledge, provided or gained, contrary to the claim made by Zhang et al., (2009). Here we test whether differences in how targets and flankers are sampled can affect crowding, even in the absence of explicit knowledge. We operationalise these differences in terms of overlap between target and flanker sets. Targets and flankers can be sampled from the same set of objects (full overlap), targets and flankers can be selected from distinct sets (no overlap), or the two sets can share some objects (partial overlap). We test if these overlap differences will modulate crowding. We will also test if the effect of category can be attributed to these overlap differences by including a condition where the flankers are chosen from a different category (and hence, by definition, do not have any overlap with the target set).

### Observers

Twenty-six observers participated in experiment 1. Three participants were excluded from the statistical analysis. Two of them showed floor effects and the other participant was presented with the wrong set of practice conditions. Participants received monetary reimbursement or course credits for their participation. All participants had normal or corrected to normal vision. Participants gave written informed consent. The study was designed and conducted under the approval of the Psychology Ethics Committee at the University of Aberdeen.

### Stimuli and material

Stimuli were generated in MATLAB using the Psychophysics toolbox extensions (Brainard, 1997; Kleiner et al., 2007; Pelli, 1997), and were displayed on a 19-inch Sony CRT-Screen with a frame rate of 100 Hz and a resolution of 1024 × 728 pixels. The viewing distance was set to 57 cm secured by the use of a chin rest.

**Fig. 1:**
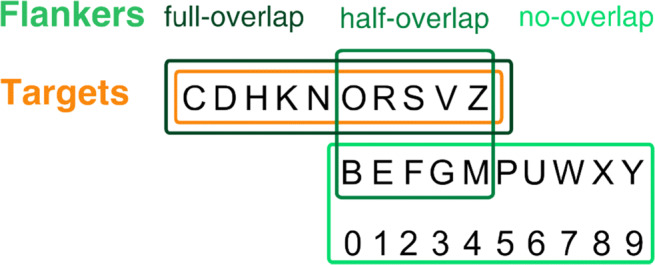
Target- and flanker-sets used in Experiments 1 and 2. The top row of letters was always the target set (orange box). The flankers could be drawn from the same set (full-overlap condition; dark-green), or from a different set of letters (no-overlap condition; light-green). The flanker set could also be made up of letters that were drawn from the two distinct sets of letters (five each, randomly chosen, not just the set pictured here; half-overlap condition; medium green)

**Fig. 2:**
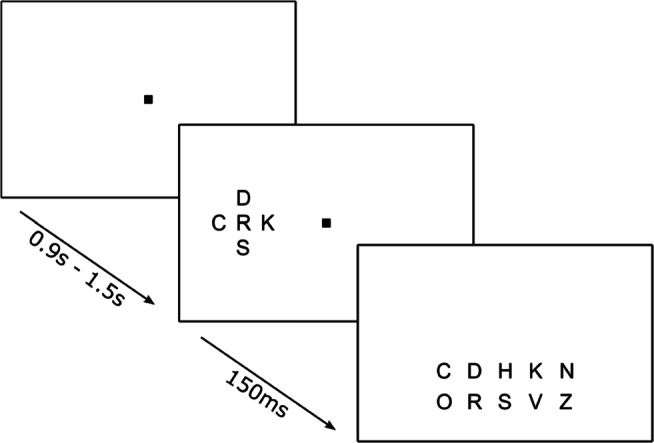
General Procedure: A fixation mark was displayed in the centre of the screen. After variable fixation intervals the target with four flankers was presented in the right or the left visual field for 150ms. 200ms after stimulus offset all possible target stimuli were shown for mouse response

Twenty uppercase letters and ten numbers served as stimuli (Fig. 1). All characters were drawn in Geneva font and subtended approximately $1.2\deg $. Stimuli were presented in black (*luminance* = 0.25*cd*/*m*^2^) on a white background (91.5*cd*/*m*^2^) and hence were presented at a high contrast (*C*_*Michelson*_ = 0.99). The stimulus display consisted of a target surrounded by four flankers, one in each cardinal direction (left, right, top and bottom; see Fig. 2). The target was presented centred on the horizontal meridian at and eccentricity of $10\deg $, either to the left or right of a centrally presented fixation mark (small square). Flankers (letters or numbers) were presented equidistant from the target (letters only).

The QUEST algorithm controlled the centre-to-centre spacing between the target and flankers (Watson & Pelli, 1983) for runs of 32 trials. Critical spacing was determined as the spacing at which performance was estimated to be at 82% (QUEST parameters: SD of the Weibull fit = 3, Slope of the psychometric curve = 3.5; Finger error rate = 0.01). To estimate critical spacing thresholds, QUEST was used to re-compute a single threshold over the pooled trials for each of the conditions, respectively. This is the equivalent of considering that all trials were derived from the same run (for example, one threshold from a run of 160 trials instead of an average of 5 thresholds obtained from 32 trials each). Hence, all trials are included, and no data is lost, which can occur if certain exclusion criteria that are often employed when traditional averaging are used are met (e.g. SD for estimated block-wise threshold is too high).

Critical spacing was assessed in four conditions differing in overlap and flanker category. The same 10 letters were used as targets in all conditions (target-set). The flanker-set was manipulated across the four conditions. In three conditions, letters were used as flankers, thus sharing their category with the targets. In the full overlap condition flankers were drawn from the same set of 10 letters as the targets. In the partial overlap condition, half the flanker letters were randomly selected from the target-set and the other half were randomly selected from the 10 non-target letters. This flanker-set was then used throughout the experiment for that participant. In the no overlap condition none of the flanker-letters were chosen from the target-set; they were the 10 non-target letters. In the final condition, target and flanker-sets differed in category – flankers were numbers; hence no overlap of stimulus sets was possible (Fig. 1). No information was provided to the participant about the conditions. Conditions were blocked. Participants took part in 24 blocks, 1 practice block and 5 experimental blocks for each of the 4 conditions. For a given trial, target and flankers were randomly drawn from the respective target and flanker-sets.

### Procedure

Participants initiated each block of the experiment with a button press, while resting their head on a chinrest. Each trial started with the presentation of the fixation mark. After a variable fixation interval (0.9 to 1.5 s), the stimulus was presented for 150 ms. The fixation mark remained on the screen for a further 200 ms after which all characters of the target-set (potential response options) were displayed in the lower half of the screen. Participants were asked to report the target via a mouse click on the appropriate character; auditory feedback was provided. The next trial started automatically after the response was given. A short break was offered after each block.

### Results

**Fig. 3:**
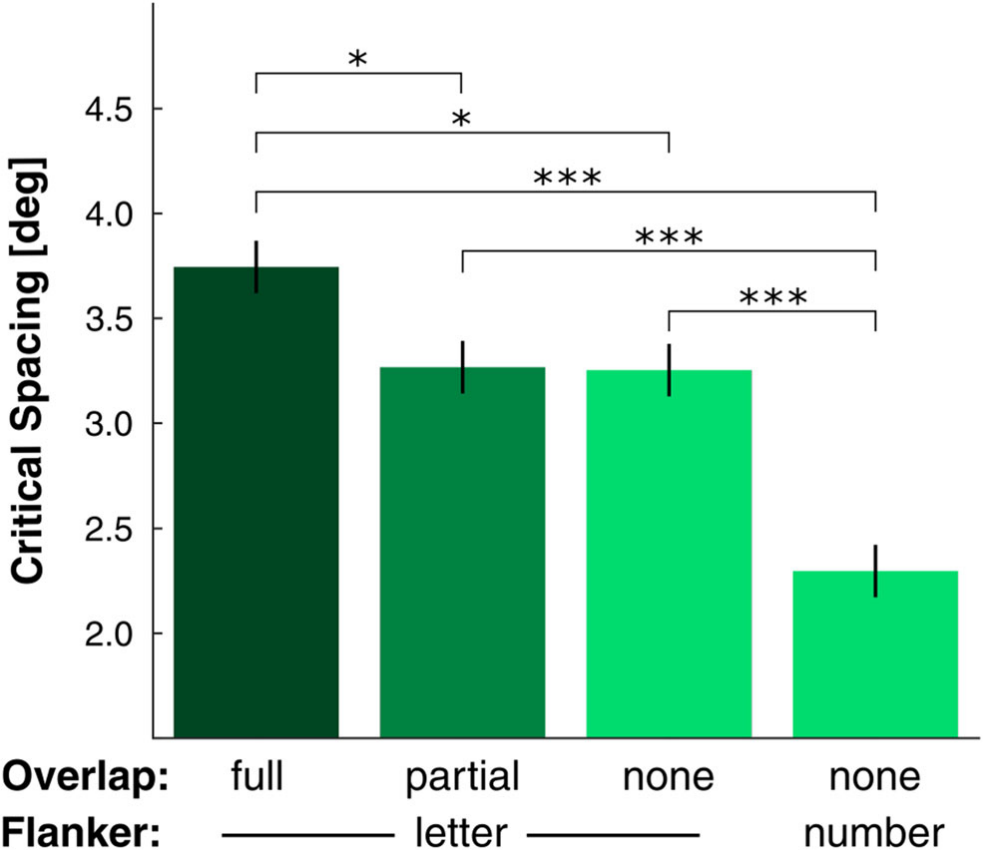
Results of Experiment 1: Critical spacing is shown for the different conditions defined by overlap (full: dark green, partial: mid-green, none: light green) and flanker type (letters or numbers). Error bars are within-subjects ± 1 SEM_L&M_

The results of experiment 1 are shown in Fig. 3. Here, we tested if crowding is modulated by differences in overlap between target- and flanker-sets when participants are not explicitly made aware of the different overlap conditions. We also assessed whether this modulation can partly or fully account for differences in crowding that have been ascribed to target and flanker categorical similarity. That is, using the effect of category membership as an example, we tested if unintentionally introduced overlap-differences can explain the results thought to be due to higher-level differences between targets and flankers.

The extent of overlap between target and flanker sets had a substantial effect on critical spacing (one-way repeated measures ANOVA: $F_{(3,66)}= 27.0; p< .001; {\eta _{p}^{2}}= .551$). Post-hoc pairwise comparisons (Bonferroni-Holm correction applied) were then conducted to determine the basis of this effect. Among conditions where targets and flankers belonged to the same category, crowding was found to be stronger in the full overlap condition compared to the partial-overlap (*t*_(22)_ = 2.66;*p* = .029) and the no-overlap (*t*_(22)_ = 2.94;*p* = .023) conditions. The critical spacing in the full overlap condition was 13.6% higher than in the partial overlap condition and 14.0% higher than in the no overlap comdition. One might have expected to find a gradual decrease in crowding from full-, to partial- to no-overlap conditions. However, no difference was found between the partial-overlap and the no-overlap condition (*t*_(22)_ = .100;*p* = .922). This might appear to contradict Zhang et al.,’s (2009) results, which did show such a difference. However, in their experiment, overlap and target-flanker similarity changed concurrently. That is, the gradual decrease in crowding reported in their study could be due to a gradual increase in target-flanker complexity differences, rather than a change in overlap.

In summary, we found crowding to be strongest when the same set of stimuli was used for target and flankers, even though our participants were not made aware of the different flanker conditions; crowding was weaker when target- and flanker-sets were fully or partly distinct. Hence, explicit knowledge about flanker-conditions, and therefore about differences in flanker-set overlap is not necessary for overlap to have an effect of crowding.

We also investigated the influence of overlap on the category effect. We found that crowding was stronger when target and flankers shared category membership (letters) than when flankers belonged to a different category (numbers), which is in line with previous studies that investigated the effect of object category and meaning (Huckauf et al., 1999; Reuther & Chakravarthi, 2014). In fact, we observed this category effect at all levels of overlap in the same category conditions (full-, half- and no- overlap letter flankers; all *ps* < .001). The individual pairwise comparisons can be seen in Table 1. Previous experiments that tested the effect of object category on crowding used stimuli with full-overlap between targets and flankers in the same-category condition (targets and flankers are chosen from the same set), whereas this is not possible in the different category condition (Huckauf et al., 1999; Reuther & Chakravarthi, 2014). The set-up of the current experiment allows us to assess the influence that differences in target-flanker-set overlap might have had in these studies. The category effect (the difference in critical spacing between same and different category conditions) was $1.45\deg $, or 48% when there was full-overlap in the same-category condition, whereas it was $0.96\deg $, or 34% when there was no overlap in the same-category condition. That is, the category effect was found to be reduced by 28% when conditions only differed in category, than when they differed in category and overlap. Therefore, controlling for overlap reduces the category effect. Previous experiments inadvertently included overlap differences as a confound. We take this to suggest that the previously observed category effect can at least partly be accounted for by overlap differences between target- and flanker-sets. Interestingly, our previous work (Reuther & Chakravarthi, 2014) also found that when featural differences were largely controlled for, the category effect was largely diminished, although not eliminated. The remaining effect (10-15%) might perhaps have been due to differences in overlap between target and flanker set in the same category condition but not in the different category condition. This finding taken with the current results suggests that the so-called category effect, which has been used to argue for multi-level crowding, might potentially be fully accounted for by featural differences and overlap differences.

**Table 1:** Pairwise comparisons with Bonferroni-Holm corrections applied in Experiment 1

Pairwise comparisons	
∙ between different overlap conditions:	
∘ full overlap compared to half overlap	*t*_(22)_ = 2.66; *p* = .029
∘ half overlap compared to no overlap	*t*_(22)_ = .100; *p* = .922
∘ full overlap compared to no overlap	*t*_(22)_ = .935; *p* = .023
∙ for the category effect:	
∘ full overlap letter flankers compared to number flankers	*t*_(22)_ = 6.92; *p* < .001
∘ half overlap letter flankers compared to number flankers	*t*_(22)_ = 8.88; *p* < .001
∘ no overlap letter flankers compared to number flankers	*t*_(22)_ = 5.71; *p* < .001

## Experiment 2: Overlap effects without implicit knowledge

In experiment 1, the overlap between the target and flanker sets modulated the extent of crowding even when participants were not made aware of the different flanker-conditions and thereby of the differences in target-flanker-set overlap. This suggests that the effect does not necessarily rely on top-down feedback. However, in experiment 1 the response options remained the same throughout. That is, although participants were not made aware of the existence of different flanker-conditions, over several trials, they could have gained and used the knowledge about the possible target identities to facilitate target-recognition. Here we tested if such potential acquisition of knowledge about target identities is necessary to produce an effect of overlap. We determined if the overlap effect can be reproduced even when such knowledge is impossible to derive. This, therefore, also tested if the effect of overlap is a higher-level effect.

### Observers

Seventeen observers participated in experiment 2. One participant was excluded from statistical analysis for showing floor effects in both no-overlap conditions (those were expected to show the weakest crowding). Participants received monetary reimbursement or course credits for their participation. All participants had normal or corrected to normal vision. Participants gave written informed consent. The study was designed and conducted under the approval of the Psychology Ethics Committee at the University of Aberdeen.

### Stimuli and procedure

**Fig. 4:**
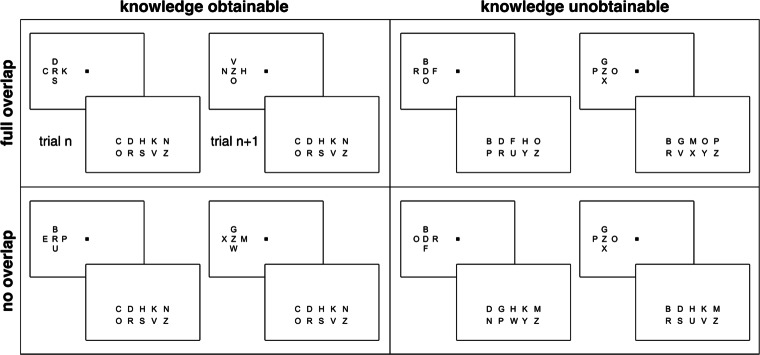
Stimuli and response options in each condition in Experiment 2. In the knowledge obtainable conditions (left column) the target-set and the response options remain the same throughout the experiment. In the knowledge unobtainable conditions (right column) they change on a trial by trial basis. In the conditions with overlap (upper row) the target and the flankers are among the response options, but in the conditions without overlap only the target is

Two factors were manipulated, target-flanker-set overlap (full or none) and knowledge (obtainable or unobtainable), leading to 4 conditions. The same 20 letters as in experiment 1 were used. The two overlap conditions where knowledge was obtainable were essentially the same as the full- and the no-overlap conditions in experiment 1. That is, the same set of 10 letters was used as the target-set for all blocks in these conditions. The flanker-set was either the same as the target set or constructed from 10 letters that did not belong to the target set. In the knowledge unobtainable condition, on each trial, 10 of the 20 letters were randomly assigned to the target-set. The flanker-set then consisted of the same 10 letters in a full-overlap trial or the remaining 10 letters in a no-overlap trial. Overlap and no-overlap conditions were blocked, to match the knowledge obtainable conditions. After each trial, all target options (10 possible responses) were displayed for selection by mouse. Note that the flankers were reportable only when they were drawn from the target-set (full-overlap condition). Figure 4 shows two example trials for each of the 4 conditions.

The experiment consisted of 24 blocks of 32 trials. Four of these blocks, one for each of the conditions, were presented at the beginning of the experiment as practice. This was followed by the main testing session, which consisted of two parts. Participants were tested on 10 blocks of the knowledge obtainable conditions in one part and 10 blocks for the knowledge unobtainable conditions in the other. The order of the parts was counterbalanced. Within each part half of the blocks (5) were full-overlap blocks and the other half no-overlap blocks. The order of blocks within a part was randomised. Apart from these differences, stimuli and procedure were the same as in experiment 1.

### Results

**Fig. 5:**
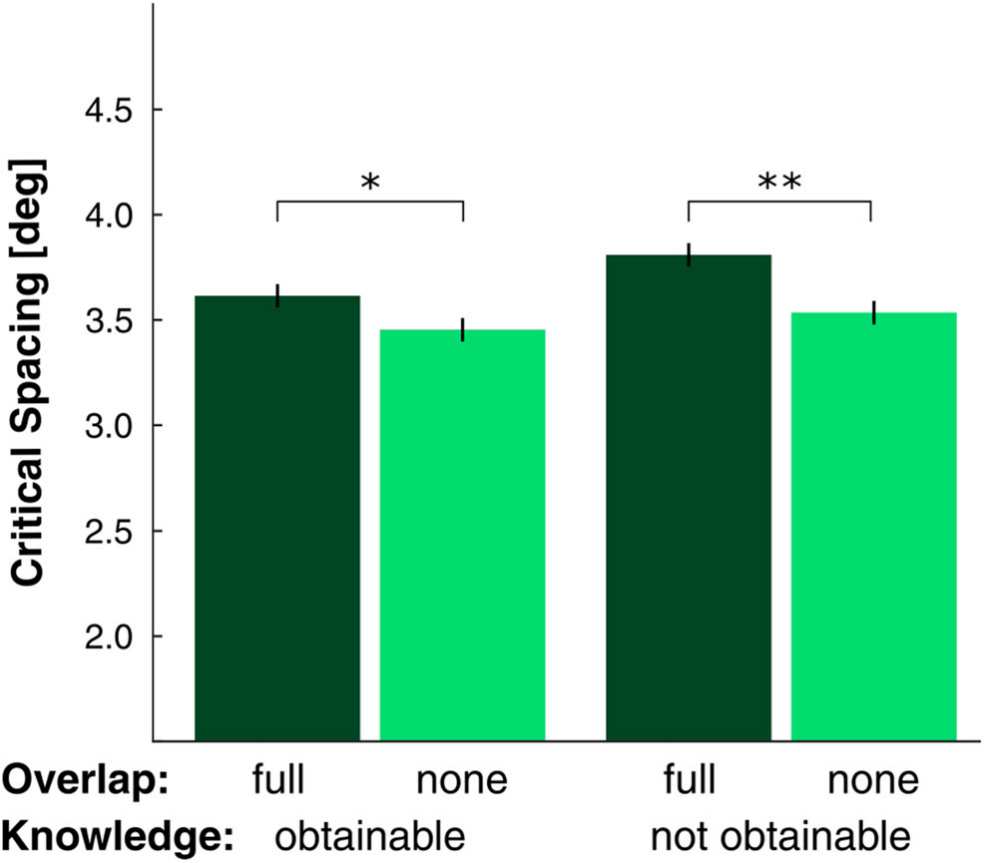
Results of Experiment 2: Critical spacing is shown for the different conditions defined by overlap (full: dark green, none: light green) and knowledge (obtainable and not obtainable). An effect of overlap (difference between full and no-overlap conditions) is present for both knowledge conditions. Error bars are ± 1 SEM_L&M_

In experiment 2, we tested if the effect of overlap, observed in experiment 1, could have been due to acquisition of knowledge about target identities and flanker conditions. If this were true, a difference due to overlap would only be expected between conditions where participants could potentially obtain such knowledge. This would only be the case in that part of the experiment in which the targets remained the same on each trial and block. However, if knowledge is not needed an effect of overlap should be observable even in the other part, where the target set changed on each trial. The results of experiment 2 can be seen in Fig. 5.

A 2 (knowledge: obtainable, unobtainable) by 2 (overlap: full, none) repeated measures ANOVA on critical spacing revealed a main effect for overlap ($F_{(1,15)}= 15.7; p= .001; {\eta _{p}^{2}}= .512$). Crowding was stronger when there was full overlap between target- and flanker-sets compared to when target- and flanker-sets were distinct. However, neither an effect of knowledge ($F_{(1,15)}= .572; p= .461; {\eta _{p}^{2}}= .037$) nor an interaction ($F_{(1,15)}= 2.46; p= .138; {\eta _{p}^{2}}= .141$) was observed. The overlap effect was observed in the knowledge-obtainable (*t*_(15)_ = 2.67;*p* = .018) as well as in the knowledge unobtainable conditions (*t*_(15)_ = 3.89;*p* = .001). In the knowledge obtainable condition, the critical spacing was 4.6% higher in the overlap conditions than the in the no-overlap condition; in the knowledge unobtainable condition, the difference was 7.5%.

These results replicate the finding that overlap between target and flanker sets alters the extent of crowding and that explicit knowledge about different overlap conditions is not necessary for this effect. Furthermore, we showed that knowledge about possible target-identities is unnecessary, as an effect of overlap was observed even when the possibility of knowledge acquisition was eliminated; yet the size of this effect was comparable across the two conditions. In these trials, target identity information from response options could only be derived after stimulus presentation. This excludes the possibility that top-down *knowledge-based* feedback influenced feature integration and therefore affected the perceptual stages of object recognition.

## An alternative account for overlap effects

Experiments 1 and 2 demonstrated that knowledge about different flanker-conditions and knowledge of possible target identities are unnecessary for overlap effects to occur. Crowding was reduced when target- and flanker-sets were distinct, even though the participants were not made aware of different flanker conditions and even if they could not obtain knowledge about possible target identities (prior to the presentation of response options after each trial). Clearly, top-down knowledge affecting the feature integration process, as proposed by Zhang et al. (2009) is not necessary.

But what could lead to the observed differences in the extent of crowding? In the experiments described by Zhang et al.,(2009) and those described above, differences in overlap imply that there are differences in flanker-reportability. When there is overlap between target- and flanker-sets, that is, when target and flankers are sampled from the same set of stimuli, then not only the target but also all flankers are reportable. Here, the flankers can potentially be misreported as targets. However, when there is no overlap, then only the target is among the response options. Flankers can never be misreported as targets. Hence, if a letter is identified and is among the response options, it can be confidently reported as the target, irrespective of the perceived location of the letter in the stimulus array. We propose, that this difference in reportability of flankers is sufficient to lead to the observed difference in crowding. According to this account, the difference in conditions arises purely at the response selection stage and not during perceptual processing of the stimuli. This explanation does not require any higher-level or top-down modulation of crowding or feature integration. We will test this proposal by developing a simple model based on the experimental parameters of our experiments and of those of Zhang et al., (2009). First, however, we will discuss the theoretical basis for the model.

Target-flanker swapping errors are common in crowding tasks. In up to half and, on average, in a third of all error trials participants reported a flanker instead of the target (Strasburger & Malania, 2013; Strasburger, 2005; Chung & Legge, 2009). Therefore, the potential for differences between conditions that do and do not allow a flanker to be reported is high. Even if only one flanker was identified, performance in conditions with and without overlap can differ. In the condition with overlap, the flanker can be reported and assuming that targets and flankers in a given trial are sampled without repetition (i.e. they can never have the same identity), this leads to an incorrect answer. In the condition without overlap, the flanker cannot be reported. Therefore, a participant might randomly pick one of the response options, which increases the probability of a correct answer from zero to chance. Note that this assumes that the observer has no information about the target. Realistically, they are likely to have some information through processes such as averaging. Hence the difference between the two overlap conditions should be even higher.

However, from experiments where participants were asked to report all stimuli instead of only the target, we know that participants are likely to identify more than one object. Even under conditions of strong crowding (centre-to-centre spacing between letters as small as $0.4\deg $ at eccentricities of $4\deg $ and higher), recognition accuracy for the individual positions of a trigram lay between 34 and 57%, when all 26 letters of the alphabet were used as targets (Huckauf & Heller, 2002). When 10 letters were presented in a circle around a fixation mark with an inter-letter spacing of around half the eccentricity, between 4 to 6 letters could be recognised correctly (Popple & Levi, 2005). That is, in commonly used stimulus settings that present a target surrounded by 2 or 4 flankers, participants are likely to identify multiple, if not all, of the presented objects. Zhang et al., (2012) analysed error-types in a full report task differentiating between location and identity errors. They found that, in 40.1% of the error trials the target was identified correctly, but was mislocalised and reported at one of the flanker locations under conditions with strong crowding (mean error rate: 64.8%). Given such frequent mislocation errors, the probability of a correct response is higher if there is no option to report any flanker. That is, if only the target can be reported, it will be reported irrespective of its perceived location. Hence, the potential difference between conditions that do and do not allow a flanker to be reported is substantial.

Using an analytical model we will compute the magnitude of performance differences between conditions with and without overlap that can be explained by the differences in flanker-reportability.

The model is based on the following premises and observations:

1. For a certain proportion of trials object position information is lost leading to a position swap between the target and the flankers. Yet, all objects are identified correctly in these trials.
2. The probability of position information loss is high when performance is low and it decreases (linearly, e.g. Strasburger & Malania 2013) with no loss of position information when targets are identified with an accuracy of 100%.
3. Object position information loss only leads to perceived position swaps between radially aligned objects (e.g. Zhang et al., (2012)). Only immediately adjacent flankers will be considered (triplet). The model can be extended to include other flankers, but most swapping errors usually occur for adjacent, radial flankers, and hence we restrict swapping errors to these.
4. When object position information is lost, performance is influenced by flanker-reportability. When only the target is reportable, it will be reported correctly. When one or more flankers are also reportable, performance depends on the chance of reporting the target among all objects that are identified and reportable (e.g. for a triplet in the full overlap condition, if all three are identified, accuracy would be one-third).
5. Performance for a condition with no overlap (only the target is reportable) will be considered as baseline performance. Conditions with overlap, e.g. all characters of a triplet are reportable, will have lower performance for the proportion of trials that are affected by object-position information loss. The reduction is based on the difference in the chance to report the correct object when position information is lost e.g. 1 when only the target is reportable, compared to a 1/3 for three reportable characters.
6. No further reductions of performance (while implementing point 5) will be allowed if performance in the no-overlap condition is already at chance.

To implement these points, we use a standard Weibull psychometric curve to describe the performance for conditions without overlap (*P*_*no*_):

1 $$  P_{no} (x,\alpha,\beta,\gamma)= \gamma+(1-\gamma)e^{-\left( \frac{x}{\alpha}\right)^{\beta}} $$

where *γ* is the chance level, $\alpha =\frac {1-\gamma }{2+\gamma }$ is the midpoint of the curve and *β* corresponds to the slope of the function.

To determine the performance for the conditions with overlap (*P*_*over*_), first results of Eq. 1 are corrected for chance using the following equation (Klein, 2001).

2 $$  P_{corr} = \frac{(P_{no}-\gamma)}{1-\gamma} $$

Next, an equation is derived to determine the proportion of trials that are affected by object-position information loss (*P*_*infl*_). Following premise 2, the proportion of swapping errors *s* is highest (*s*_*max*_) when identification performance in the no-overlap condition is lowest (*P*_*low*_) and linearly decreases with an increase in performance, with the least proportion of swap errors (*s*_*min*_) at maximum performance (*P*_*high*_). For example, 40% of trials might have swapping error when performance is just above chance, and this percentage of trials with swapping errors linearly decreases to 0% as accuracy increases to 100%.

This change in proportion of trials with swapping errors takes the form of a linear equation (*y* = *mx* + *c*), where *x* is *P*_*corr*_, *c* is *s*_*max*_ and *m* is based on the gradient between the points *P*_(*x*1, *y*1)_ = [*P*_*low*_, *s*_*max*_] and *P*_(*x*2, *y*2)_ = [*P*_*high*_, *s*_*min*_].

3 $$  P_{infl} = \frac{s_{min}-s_{max}}{P_{high}-P_{low}}*(P_{corr}-P_{low})+s_{max} $$

We take the values of *P*_*low*_ and *s*_*min*_ to be 0; Eq. 3 therefore reduces to:

4 $$ P_{infl} = \frac{s_{max}*(P_{high}-P_{corr})}{P_{high}} $$

The difference between performance in conditions with (*P*_*over*_) and without overlap (*P*_*no*_) depends on the difference in the probability (*p*_*diff*_) of reporting the target correctly in the trials where position information loss occurs (*P*_*infl*_) in the two overlap conditions (*p*_*no*_) and (*p*_*over*_).

5 $$  p_{diff} = p_{no}-p_{over} $$

In both conditions (with and without overlap), the probability (*p*) of reporting the target (*T*) correctly depends on the number of reportable flankers (*F*_*n*_) and, given that the flankers are reportable, the stimulus sampling technique used: a) with^1^ and b) without replacement.

$$ p=P(A|B) = P(A)* P(B) $$

where *P*(*A*) is the cumulative probability with which each of the presented objects (target and flankers) assume the identity of the target.

$$ \begin{array}{@{}rcl@{}} P(A)  &=& P(A_{T})+P(A_{F1})+{\cdots} +P(A_{Fn})\\ &&{\kern6.1pc} \qquad with: \qquad P(A_{T})  =  1 \\ &&{\kern5.3pc}\qquad and  a): \qquad P(A_{F})  =  \gamma \\ &&{\kern6.1pc}\qquad or b): \qquad P(A_{F})  =  0 \end{array} $$

and where *P*(*B*) is the probability with which a given object is reported.

$$ P(B) = \frac{1}{1+Fn} $$

Hence, in conditions without overlap (*F*_*n*_ = 0), the probability for a correct target report is *p*_*over*_ = 1. In conditions with overlap (*F*_*n*_ = 2), the probability is a) $p_{over}=\frac {1+F_{n}*\gamma }{(1+F_{n})}$ when stimuli are sampled with replacement or b) $p_{over}=\frac {1}{1+F_{n}}$ when stimuli are sampled without replacement. Consequently the equations for *p*_*diff*_ are:

$$ a)  p_{diff} = 1-\frac{1+F_{n}*\gamma}{1+F_{n}} $$

$$ b)  p_{diff} = 1-\frac{1}{1+F_{n}} $$

Finally, Eqs. 1, 2, 4 and 5 are combined to obtain performance in the condition with overlap.

6 $$  P_{over} = P_{no}-P_{corr}*P_{infl}*p_{diff} $$

This gives us performance as a function of spacing in the overlap and no-overlap conditions. We extract critical spacing from these curves (defined as the spacing between the target and its flankers at which target identification accuracy corresponds to the threshold that was applied by the respective studies: 70.6% for the data of Zhang et al., (2009) and 82% for the data of the current study). We then compute the reduction in the extent of crowding in the no-overlap condition relative to the overlap condition.

**Fig. 6:**
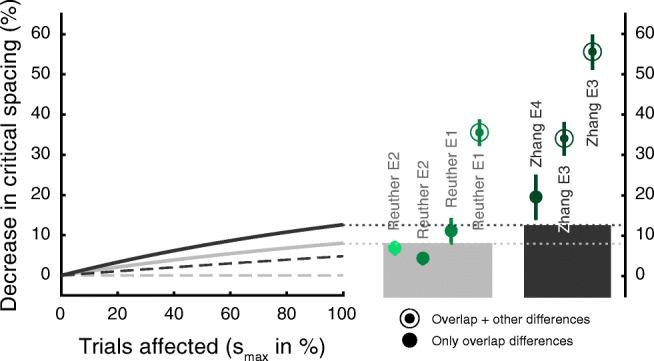
Modelling the effect of overlap: Curves depict the reduction in crowding (percent change in critical spacing) in the no-overlap condition relative to the full-overlap condition, as predicted by our analytical model. The various curves are model outcomes derived on the basis of parameters in our and Zhang et al.’s experimental designs (grey and black curves, respectively). The differences are larger when assuming that the target is among the identified objects (solid lines) than when assuming that only a flanker has been identified (dashed lines). Grey bars indicate the maximal predicted change (dark grey: Zhang et al.; light grey: our experiments). Observed experimental effects of overlap reported by Zhang et al. and our studies are indicated by circles. Error bars indicate the SEM. Filled circles (column 1-3 and 5) represent the conditions where only overlap differed between target and flanker conditions. Conditions represented by annuli had additional differences: in stimulus category (4) or complexity (6-7). The lightness of the circles indicates the extent of knowledge that participants had or could obtain about the different conditions (dark green: explicit knowledge; mid green: knowledge obtainable; light green: knowledge not obtainable). The experimental conditions that only manipulated overlap (1-2) are well described by our model, which only takes overlap into account, suggesting that overlap effects can be explained without recourse to top-down modulation of perceptual processes. It also indicates that additional factors are at play in the other experiments

Figure 6 shows the difference in the extent of crowding due to differences in overlap (full vs. none) as described by the analytical model (curves and shaded areas) as a function of the percentage of error trials where target-flanker swaps (*s*_*max*_) have occurred. Also shown are the empirical data from Zhang et al., (2009)’s study (circles with black labels) as well as from the current study (circles with grey labels). Differences are expressed as a reduction in crowding (or an improvement in performance) in conditions with no overlap compared to conditions with overlap.

The decrease in crowding is most pronounced when, in addition to differences in overlap (filled circles), targets and flankers also differ in some other way, such as in their complexity or category (annuli). Depending on the number of trials affected by swapping errors (determined by *s*_*max*_) and the number of response options, the model predicts reductions in critical spacing of up to 8.0% and 12.6% for our and Zhang et al., (2009) experimental set ups, respectively. The predicted changes are larger if the target is among the identified objects, than when only a flanker has been identified (solid compared to dashed lines). As can be seen in Fig. 6 the model accounts well for the data of experiment 2, where only letters were used as targets and flankers, and participants had no explicit knowledge of the different flanker conditions. For these conditions, object position information loss would need to affect approximately between 45% and 80% (*s*_*max*_) of the trials when performance is just above chance to account for the behavioural findings. These findings indicate that a bottom-up model that takes only overlap differences into account is sufficient to explain the results, without invoking top-down influences on crowding.

**Fig. 7:**
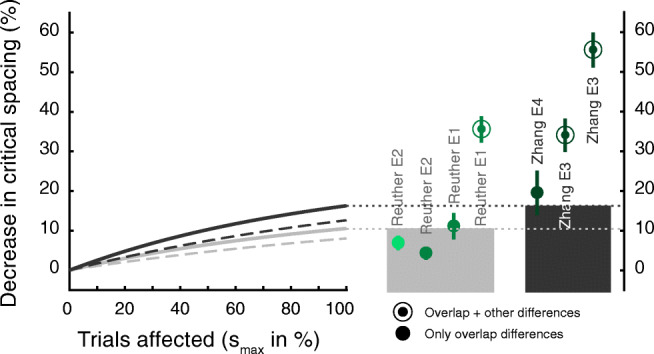
Modelling the effect of overlap: Curves depict the reduction in crowding in the no-overlap condition relative to the full-overlap condition, as predicted by our analytical model. Here, the curves (solid) are derived on the basis that the observer always reports the object that was perceived in the central (target) location, instead of reporting one of the presented objects at random as it was assumed for the conservative model (results plotted for comparison, dashed lines). Since position swaps between the target and one of the flankers are presumed for all of the affected trials, reporting the centrally perceive object leads to more errors in the condition with overlap. This model accounts for all the observed effects of overlap reasonably well (filled circles)

On the other hand, there are experimental results that the model does not capture well. The conditions where knowledge is obtainable in experiment 2 are physically identical to the full- and no-overlap conditions in experiment 1. However, the model only just accounts for the overlap effect in the latter (Fig. 6, column 3, from the left). Further, it fails to account for the observed difference in crowding when target-flanker category similarity (e.g., when letter targets are surrounded by number flankers) is added to the mix (Fig. 6, column 4). Similarly, the model cannot account for the Zhang et al., (2009) data (Fig. 6, columns 5-7), where participants were provided with explicit knowledge about the flanker conditions and the possible target responses, particularly when target and flankers also differed in character complexity in the no overlap condition (annuli). However, our model is conservative. It presumes that participants report one of the presented objects at random when position information is lost and a position swap between the target and one of the flankers occurred in the full-overlap condition. Instead, the model data depicted below (Fig. 7), is based on the premise that, as instructed, participants report whichever object was perceived in the central (target) location in the overlap condition. This consequently leads to an error, but might be closer to actual behaviour, since it is unlikely that the participant has any insight into whether a position swap occurred or not. Under this premise the model accounts for reductions in critical spacing of up to 10.6% and 16.3% for our experimental design and for that of Zhang et al., (2009), respectively. It accounts for Experiment 1’s data in our study and also the data in Zhang et al.’s study sufficiently well when overlap is the only difference between conditions (filled circles). Again, this model fails to account for the data where conditions differed also in similarity of either category or complexity (annuli), or where participants were provided with knowledge about flanker-reportability (dark green circles). Therefore, in addition to the changes that might originate from differences in flanker-reportability, there must be other mechanisms that make use of these differences in target-flanker similarity and knowledge. We will describe possible ways of implementing these in the discussion.

## Discussion

In this study, we demonstrated that crowding can be directly influenced by differences in target-flanker sampling. Crowding was found to be stronger when both target and flankers were drawn from the same set of stimuli (with overlap), compared to when they were drawn from different stimulus sets (without overlap).

Explicit knowledge about differences in target-flanker-set membership (i.e. overlap) and about possible target identities has previously been suggested as the source of these differences (Zhang et al., 2009). Here, we found that explicit knowledge about differences in overlap is not necessary (experiment 1 and 2). Further, the availability of information about possible target identities prior to stimulus presentation (experiment 2) is also not required to elicit this effect. This rules out top-down knowledge as the source of the effect of overlap.

An alternative source for the effect of overlap is the difference in flanker reportability introduced by differences in overlap in experiments with restricted response options. That is, in a condition with overlap, all stimulus objects can be reported. However, in a condition without overlap, only the target can be reported. This lack of flanker-reportability can be used to identify the target, prevent flanker errors and, as a consequence, lead to weaker crowding. We implemented an analytical model to test the claim that a difference in flanker-reportability can lead to differences in crowding. The model demonstrated that differences in flanker-reportability alone might account for a change in critical spacing (extent of crowding) by around 10% (depending on the underlying chance level).

If this account were the root cause of the effect of stimulus-set overlap, it would naturally predict that the effect would fail to appear if the response options were unrestricted. Since this would allow for flanker-reports in both the overlap and the no-overlap condition similarly, it would equate performance. However, when considering the implementation of such an experiment, we should keep a couple of caveats in mind. The effect of an unrestricted response-set can be tested in one of two possible settings: a) when knowledge is impossible to obtain, as in the current Experiment 2, or b) when knowledge could potentially be obtained. In the former case, a null result would not confirm the proposed account. This is because if stimuli are randomly assigned to target- and flanker-sets on a trial-by-trial basis *and* if the responses are unrestricted, it would in practice obliterate stimulus-set membership altogether. Here, the target-set is revealed to the observer only through the set of response options that is provided at the end of each trial. Hence if all letters are reportable, then it is not possible to separate out the flanker- and target-sets. On the other hand, testing the above proposal when target- and flanker-sets are kept constant across trials, that is when knowledge is potentially acquirable, is also not informative. Finding a difference in crowding between the two overlap conditions in such an experiment would not imply a rejection of our proposal. In this setting, any acquired knowledge about the stimulus-sets would likely lead participants to restrict the set of used responses despite a lack of these restrictions being imposed by the experiment. Nevertheless, we conducted this version of the experiment (please see Supplementary Materials) and found that, once again, crowding was stronger in the overlap condition than in the no-overlap condition. Further, a substantial number of observers could, when prompted, correctly point out the set of target letters or at least a large majority of this set. This indicates that over the course of the experiment they could explicitly determine which letters belonged to the target set, which would have helped them perform better in the no-overlap condition. Hence, rather than refuting our proposal, this could be seen as indirect evidence for the role of flanker-reportability.

Yet, it is unlikely that the difference in flanker-reportability is the only factor that modulates the differences observed in such experiments. The reported magnitudes of differences in the extent of crowding between conditions differ vastly across experiments and can easily exceed the model predictions by 2 to 6 times. Hence, below, we propose a mechanism for the effect of overlap that takes into account various factors, besides flanker-reportability, such as top-down knowledge and incidental differences between the target- and flanker-sets.

### Flanker-reportability

The mechanisms underlying the effect of overlap, where crowding is reduced when target- and flanker-sets are distinct compared to when they are not, can be partly explained as being due to the consequence of position information loss under crowded conditions. Position information loss is one of the sources that has been proposed to underlie visual crowding (e.g. Ester, Klee, & Awh, 2014,2014; Greenwood et al., 2009; Hanus & Vul 2013; Nandy & Tjan 2007; Parkes et al., 2001; Strasburger & Malania 2013). It can be subdivided into two forms based on its consequences (Strasburger et al., 2011): Position information loss can affect the individual features of objects either jointly or separately. If features are affected jointly, objects retain their global shape but lose their local position information (object position information loss). This can lead to perceived position swaps between target and flanker objects and in turn to positional errors, where an observer reports a flanker instead of a target. If, instead, position information loss affects features separately (feature position information loss), objects lose their global shape and features of different objects can be combined together. This can than lead to identity errors, where an observer reports an object that was not part of the stimulus display.

If there is no overlap between target- and flanker-sets, the effects of object position information loss can be alleviated. In this situation, a target object can be accurately reported even if its precise location is unknown, since no other identified object is reportable. Whereas, in conditions with overlap, all stimulus objects are reportable and the consequences of object position information loss become obvious. The size of the overlap effect is thereby dependent on the proportion of trials in which a participant reports the target correctly, even though it was not perceived in the target location.

However, if feature position information is lost, correct stimulus identification is impaired and the target will not be reported correctly, even if it is the only reportable object. In these cases, participants will likely rely on noisy template matching by choosing the response option closest to the object perceived in the target location. Therefore, one factor that can influence the likelihood of a correct target report is the ratio of trials where position information loss occurs over whole objects versus where it occurs for individual features. A larger overlap effect is expected when the ratio between object and feature position information loss is in favour of object position information loss.

### Knowledge

Although an overlap effect was observed even in the absence of acquirable knowledge, differences in knowledge might still modulate the overlap effect. This is indicated by the finding that the overlap effect was smaller when knowledge about target-flanker sets and target identities was unavailable (experiment 2, current study) compared to when it was available (experiment 4, Zhang et al., (2009)). However, rather than influencing target-flanker-segregation on the perceptual level (as suggested by Zhang et al., (2009)), knowledge is likely to influence observers’ response strategy at the level of decision making. That is, the knowledge that flankers are not reportable could increase the likelihood with which an observer reports the target even if it was not perceived in the target location, instead of choosing the response option that resembles the object perceived at the target location. As already stated, this leads to a stronger decrease in the extent of crowding in the condition without overlap and in turn, to a larger overlap effect.

Evidence that knowledge could influence the extent of the overlap might be evident in the differences in magnitude of the overlap effect between experiment 2 and experiment 1 of the current study. In our second experiment, where we found the smallest effects of overlap, our participants had no explicit knowledge about differences in flanker-reportability and were unlikely to acquire such knowledge during the course of the experiment. In the first experiment the effect of overlap was larger. Intriguingly, this was the case even in the condition that was identical to one set of conditions in experiment 2. That is, with identical stimuli and protocol, we observed differing strengths of the overlap effect. This could just be due to individual differences between the two groups of participants. However, in the first experiment, participants might have acquired knowledge about differences in reportability between target and flankers over several trials. This could have been facilitated by the condition that used numbers as flankers. There, although instructed to pay attention only to the target-location, participants might have perceived one of the number-flankers. From this they could have derived the knowledge that at least in some of the blocked conditions the flankers were not reportable. This would not have been possible in experiment 2. This difference might have driven the difference in overlap effects across the two experiments. In fact, it is not unlikely that participants acquire and apply knowledge about which stimuli do, and which do not, belong to the target-set in settings where target- and flanker-set remain the same. In a follow-up experiment that was designed to test the effect of unrestricted responses, a number of participants reported to have made use of their observation that only some of the 26 letters of the alphabet were ever presented in the target-location. This was accompanied by the finding that crowding was weaker in the no-overlap condition compared to the condition with overlap, and further that the difference was not present during training, but emerged and stabilised subsequently (see Supplementary Materials). This supports the idea that gaining knowledge can modulate response selection and consequently the extent of crowding.

However, the biggest difference in the extent of crowding between conditions with and without overlap was observed in the study by Zhang et al., (2009). In their study, unlike in ours, the number of flankers (2) and response options (5) was comparatively low; further, in the experiment of Zhang et al. participants were provided with explicit knowledge of whether the flankers in a given block of trials were reportable or not; they were also provided with a hard copy of the response options. On the other hand, in our experiment, participants were not made aware of the different flanker conditions; response options were only provided after stimulus presentation; and the number of flankers per trial (4) and the number of response options (10) was comparably high. These differences in the experimental choices, especially in the extent of knowledge provided in advance, may support the use of different response strategies.

In our study, participants were instructed to attend the target location and to report the letter that was perceived there. Since reliable information about the possible target identities became available only when the stimulus was replaced with the response options, intentional use of any other strategy would have been discouraged. That is, since the number of response options was relatively high (10) and response options were variable, it would have been unlikely that participants actively chose to remember their identities in order to make use of this information during stimulus presentation. Furthermore, to make use of the identity information after stimulus presentation, it would have been necessary to store the entire stimulus percept in visual working memory. Even though it has been suggested that this is possible when target-flanker distances are too small for object individuation (Bacigalupo and Luck, 2015), we consider this unlikely to be applied as an active strategy. In our study participants may have therefore not actively applied an additional identification strategy.

In the experiments reported by Zhang et al., (2009) the target was also defined by its position. However, given the characteristics of the experiment, the perceived object position was not the only way to correctly report the target. Participants’ may have used their knowledge about the possible target identities as an additional determinant to distinguish the target from the flankers. Based on the knowledge provided about the target identities (5 options) and the knowledge about the reportability of the flankers, we suspect that participants were aware that identification by excluding non-reportable characters (independent of their position) was a viable way to identify the target. That is, they may have used both means of identification (position and identity) actively and strategically, or might even have relied mainly on the latter. Together, this might explain the bigger effect of overlap reported in this study.

Explicit knowledge could alter performance through a complementary route. In trials where crowding is strong, the target and flankers might have been averaged or their features would have migrated (feature position information loss), explicit knowledge of target identities might help. In these trials, observers might ordinarily report the identity (template) that most closely matches the averaged percept, which would lead to errors. However, if the set of target identities is known, the object within the target-set that best matches the identities within this restricted set would be chosen, and hence this error can be minimised. That is, the erroneous template matches can be excluded leading to better performance and hence weaker crowding.

In summary, we suggest that, even though the root cause of the overlap effect lies in the difference in flanker reportability itself, knowledge about such a difference may additionally strongly modulate the magnitude of the effect and thereby the distance over which crowding seems to influence object recognition. However, rather than knowledge affecting perceptual processing, we put forward changes in (reporting) strategy, driven by explicit knowledge, as the source of the difference in the magnitude of the overlap effect between our study and that of Zhang et al.,(2009).

### Incidental differences

Another factor that seemingly contributes to the overlap effect observed in our study and in the experiments conducted by Zhang et al., (2009) are incidental differences between targets and flankers that co-occur with the differences in stimulus-set overlap. It is well known that crowding is weaker when target and flankers are dissimilar at the feature level (e.g. Chung et al., 2001; Kennedy & Whitaker 2010; Kooi et al., 1994; Nazir 1992; Põder & Wagemans 2007) or even at a higher level (Louie et al., 2007; Reuther & Chakravarthi, 2014). In experiment 1 of the current study, the difference in crowding was most prominent when targets and flankers not only differed in stimulus-set membership but also in their categories (full-overlap letter-flankers versus no-overlap number-flankers). In fact, for the same set of target-letters the difference in crowding was 51% higher when differences in stimulus-set membership and differences in category co-occurred. Similarly, Zhang et al., (2009) reported larger differences in crowding when, in addition to stimulus-set membership, target and flankers differed in character complexity. That is, for the same set of low-complexity targets, the effect of overlap appeared to be almost twice as big when the differences in overlap coincided with a difference in flanker complexity, than when overlap was the only difference. In both these cases, the increased difference is not the (direct) consequence of differences in stimulus-set overlap. Instead the increase can be traced back to, or is the direct result of low-level featural differences between the stimulus-sets. Crowding between letters and numbers has previously been shown to be modulated by featural differences, where letters presented in a natural font were more similar to other letters than to numbers and vice versa (Reuther & Chakravarthi, 2014). In a similar way, complexity influences the number of features present in each object and its brightness, and different complexities alters the similarity between them leading to weaker crowding when similarity is low. These factors are distinct from the overlap effect, but when present add to it, as targets and flankers will necessarily differ in the no-overlap condition, but not in the full-overlap condition.

Although top-down knowledge also appears to modulate crowding, neither our study nor Zhang et al.,’s (2009) study provides unequivocal evidence to conclude that such top-down knowledge modifies perceptual processing (feature integration). Knowledge might act by modifying a participant’s strategy or decision-making. At least on its own, we can exclude the possibility that such knowledge is necessary to drive the difference between conditions. Further studies are needed to isolate direct effects of top-down knowledge beyond the effects on strategy, such as on effects of target-flanker similarity, differences in overlap, and differences in reportability.

### Lessons for experimental designs

Even though top-down processes are not necessary in explaining effects of similarity on visual crowding, differences in group-membership that can co-occur with (other) similarity differences should be excluded when designing an experiment as they are likely to inflate the actual effect of interest. Here, in experiment 1, we showed that 28% of an effect that might otherwise solely be attributed to object category as the underlying source, was instead partly the result of differences in stimulus-set membership. This indicates that both our previous study (Reuther & Chakravarthi, 2014) and the study of Huckauf et al., (1999) overestimate the effect of object category on crowding.

One way to ensure that differences in stimulus-set membership have no influence on the (similarity) effect in question, is to either choose targets and flankers from the same group (e.g. letters) or to modulate stimuli only along the feature of interest (e.g. spatial frequency, polarity or colour), while at the same time ensuring that in all flanker-conditions target and flankers share the attribute (not necessarily its expression) the response aims at (e.g. all are oriented grids). This is the case for a number of frequently cited studies on the effects of feature similarity on crowding (e.g. Chung et al. (2001), Kooi et al. (1994); Põder & Wagemans (2007)).

For comparisons that rely on the use of multiple flanker-sets that are likely to differ in more than one attribute, differences in stimulus-set membership between flanker-sets can be excluded by ensuring that none of them have overlap with the target-set. Additionally, the same principle can and should be extended to the selection of the type of flankers used to test crowding. That is, when comparing the effects of different types of flankers on a given type of target, it should be ensured that only the target possesses the attribute (say a T-junction) that is essential to the respond or, if this is not possible, response options should be restricted to the target-set. Of course, another alternative is that all types of objects should share the same attributes. A number of previous studies on (similarity) effects that manipulated either low-level factors like shape and complexity, or high-level factors like object category, as well as studies that compared crowding at multiple levels, seem to violate these criteria and might have overestimated the effect of the factor of interest, whereas others seem to not have run afoul of these recommendations. For example, Nazir (1992), asked participants to report the orientation of a Landolt C target flanked by either rings, bars or tumbling E’s. The lack of a gap in the ring-flankers is fortunate as they do not share the diagnostic attribute of the target, namely the gap, while retaining overall shape similarity, putting them on an equal footing as the bars and E’s they were compared to (dissimilar objects that don’t possess the diagnostic feature either). If the study had used Landolt Cs as flankers, then these would have shared set-membership (and hence the diagnostic feature) with the target and thus the comparison across the different flanker types would have been unequal. That is, there would not only be a difference in similarity across flanker types, but also a difference in whether they possess the diagnostic feature (and set-membership). However, there are other studies that inadvertently conflate these factors with the factor of interest (Kimchi & Pirkner, 2015; Kooi et al., 1994). There, crowding was stronger when target and flankers belonged to the same group and possessed the attribute the response relies on, than when only the target did. So, it is not clear whether the observed effect was due to the factor they had intended to manipulate or the confound that the flankers sometimes share target diagnostic attributes and sometimes don’t. For example, Kimchi & Pirkner (2015) found that crowding was stronger when a square shaped target (consisting of either four lines or four L’s) was surrounded by closed squares, than when it was surrounded by flankers shaped like the features it was made of, but in a non-square arrangement. Rather than demonstrating multi-level crowding between feature and objects, this effect might be explained by differences in stimulus-set membership (square or not-square). However, the explanation might be even simpler. The effect can also be explained by the fact that the attribute the response is based on (orientation of square) in the across-level condition is present in the target (square made of individual features) *and* the flankers (closed square), but not in the flankers (features not arranged in squares) in the same-level condition. Similarly, Kooi et al., (1994) found crowding to be stronger when participants were asked to report the orientation of a T-target when it was surrounded by differently oriented T-flankers (shared set-membership), than when it was surrounded by oriented bars (different set-membership). Furthermore, based on a follow-up experiment comparing T-flankers and H-flankers and not finding a difference in crowding, they concluded that the weak crowding for the bars might be the result of them lacking a T-junction, which is the attribute that is central to the judgment of a letter T’s orientation. Hence it is important to ensure that either all objects share the diagnostic feature or that none of the various flanker types share them with the target.

Taken together, differences in stimulus-set membership and flanker-reportability between different conditions should be avoided, since both are likely to inflate or even produce a difference in crowding that might then be falsely attributed to the putative independent variable of interest.

## Conclusion

Our findings show that top-down knowledge of group membership is not necessary to elicit differences between conditions that differ in group membership. Crowding was observed to be stronger when target and flankers were sampled from the same set of stimuli (with overlap) than when they were sampled from different stimulus-sets (without overlap) even in the complete absence of knowledge prior to the presentation of response options. Based on these findings we conclude that the effect of overlap is due to the difference in flanker-reportability that is implied by differences in stimulus sampling. The effect of overlap might partly explain the effects attributed to crowding in higher levels of visual processing. The difference in reportability leads to a difference in the observed magnitude of crowding by influencing target selection at the stage of decision making.

*Neither the data of experiment*1*or*2*are available, since consent given at the time of data collection lacked a corresponding statement. Only the experiment in the supplementary material was registered on the Open Science Framework* (https://osf.io/xchya/).

## Electronic supplementary material

Below is the link to the electronic supplementary material.

(TEX 14.0 KB)

## Footnotes

1 In this case, *P*(*A*) can be greater than 1, as *P*(*A*_*T*_) and *P*(*A*_*F*_) are disjoint events.
